# Implementing an evolutionary framework for understanding genetic relationships of phenotypically defined insect biotypes in the invasive soybean aphid (*Aphis glycines*)

**DOI:** 10.1111/eva.12084

**Published:** 2013-07-24

**Authors:** Jacob A Wenger, Andy P Michel

**Affiliations:** Department of Entomology, The Ohio Agricultural Research and Development Center, The Ohio State UniversityWooster, OH, USA

**Keywords:** adaptation, insect biotype, plant resistance, population genetics, soybean aphid

## Abstract

Adaptive evolution of pest insects in response to the introduction of resistant cultivars is well documented and commonly results in virulent (*i.e*., capable of feeding upon resistant cultivars) insect populations being labeled as distinct biotypes. Phenotypically defined, biotypes frequently remain evolutionarily indistinct, resulting in ineffective application of virulence control measures and shorter durability of resistant cultivars. Here, we utilize an evolutionary framework to discern the genetic relationship between biotypes of the soybean aphid (*Aphis glycines,* Matsumura). The soybean aphid is invasive in North America and is among the most destructive pests of commercial soybean on the continent. Attempts to breed host-plant-resistant soybean have been hampered by the emergence of virulent aphid biotypes that are unaffected by the plant's resistance mechanism(s). Comparative population genetic analysis of virulent and avirulent (*i.e*., unable to feed on resistant cultivars) biotypes found populations to be genetically indistinguishable across biotype and geographic distance, with high rates of interpopulation immigration and admixture. The lack of genetic distinction between biotypes coupled with elevated genotypic diversity within all populations suggested virulence has a nongenetic-based or includes a gene complex that is widely distributed throughout soybean aphid populations, which undergo regular dispersal and unimpeded sexual recombination.

## Introduction

The interactions between phytophagous insects and their respective host plants have long served as a model system for coevolution (Tilmon 2008). The reciprocal adaptations occurring between populations in parasitic or mutualistic relationships results in greater specificity and interdependence between species (Thompson 1994). Many naturally occurring examples exist, though the development of crop cultivars exhibiting natural pest resistance (Painter 1968; Panda and Khush 1995; Smith 2005) provides a widespread anthropogenic microcosm of this interaction. The increased selection pressure associated with the extensive implementation across landscapes accelerates insect and host coevolutionary interactions within agroecosystems as compared to natural environments. The selection pressure caused by resistant cultivars favors the evolution of virulence (*i.e*., insect populations capable of feeding and/or reproducing on resistant cultivars, Gould 1991, 1998; Rausher 2001), and ultimately decreases agricultural production and the durability of resistance.

The genetic and environmental mechanisms of pest virulence to resistant cultivars are often ill defined. Within crop resistance literature, virulent pests are most commonly organized within the pseudo-taxonomic category of biotype, an intraspecies taxon defined by a shared differentiating phenotype (Claridge and den Hollander 1983; Diehl and Bush 1984). The biotype category is commonly intended as a temporary taxonomy to be used prior to uncovering the evolutionary mechanism of the differentiating phenotypic trait. However, most biotypes fail to be reclassified and languish within these ambiguous categorizations (Downie 2010). This casts uncertainty upon the evolutionary relationships between the differentiated phenotypes, restricting the use of applied evolutionary theory to maximize the lifespan of cultivar resistance. For example, if virulent biotypes are distinct populations or evolutionary lineages, then resistant cultivars can be strategically deployed where the frequency of such virulence is low. In this study, we apply a population genetic framework, based on the theoretical work of Diehl and Bush (1984), to clarify the evolutionary relationship of a recently emerged insect pest biotype.

### The biotypic framework

The biotypic framework, as used within taxonomy, evolutionary and applied biology, is fraught with contention and ambiguity (Claridge and den Hollander 1983; Diehl and Bush 1984; Downie 2010). For the purposes of this study, we shall define the biotype as an intraspecific classification, segregating individuals by a divergent phenotypic response to an ecological variable. This definition is intentionally broad as many ecological variables have been used to assign biotype status within populations including virulence to resistant cultivars, host-plant association, pesticide resistance, virus transmission, invasiveness, and sex ratio at birth (Montllor et al. 1983; Kim et al. 2008; Peccoud et al. 2009; De Barro et al. 2011). Indeed, there are some examples of lineage specific biotypes, such as the host races of *Acyrthosiphon pisum* (Via 1999; Peccoud et al. 2009) and the *Bemisia tabaci* species complex (De Barro et al. 2011), but these represent only a subset of all biotypes and are not reflective of all evolutionary mechanisms of virulence. Rather, these examples should be organized within more appropriate evolutionarily defined taxonomies outside of biotype such as the presence of reciprocal monophyly (Via 1990).

Criticisms of the biotype taxonomy are numerous (Claridge and den Hollander 1983; Downie 2010) but commonly root themselves in the concept's inherent ambiguity, misrepresentation of adaptive evolution, and the disconnect between divergent phenotypes and genetic differentiation. Biotype falls short of being a relevant biological taxonomy because it is designated phenotypically without implication of evolutionary origin. Phenotype is the product of the interplay between genetic variation and environment and does not necessarily denote common descent. At the intraspecific level, where biotype is utilized, differential phenotypes are likely representative of transitory adaptive evolution within the population. Thus, differentiating phenotypes could be explained through a number of mechanisms outside of a novel lineage, including the utilization of latent genetic variation and nongenetic plastic effects. While common descent is not required within a taxonomic framework, it is key to avoiding artificial indices and maintaining predictive power within biological systems (Downie 2010). A lack of monophyly is of particular concern in biotype research where investigators are commonly expected to recommend strategies to manage the frequency of virulence, which may involve manipulating environmental variables to decrease selective pressure favoring virulence (Onstad 2007). Without a clear understanding of the relationships between biotype populations, the strategies utilized may be unsuitable for the targeted biotype, causing ineffective control at best and inadvertently favoring virulence at worst.

As an intraspecific taxonomy, biotypic differentiation occurs at the population level, which can then alter gene flow, selection, and structure among populations. If the diverging phenotype is genetically based, then the evolution of virulence would produce different signatures within the population, measured by the partitioning of molecular variance. Population genetic analysis allows an indirect measure of these changes through the comparison of allele frequencies and genotypic diversity within and between biotype populations while controlling for geographic-based genetic variation. Through the rapid generation of molecular markers, even in nonmodel organisms (Baird et al. 2008; Davey et al. 2011; Ekblom and Galindo 2011), we can now use modern population genetics to revisit the evolutionary-based framework suggested nearly 30 years ago by Diehl and Bush (1984). The Diehl and Bush framework removes the adaptive biotype from its current pseudo-taxonomy and places it within one of five evolutionarily relevant categories: (i) nongenetic polymorphism, (ii) polymorphic or polygenic variation within populations, (iii) geographic races, (iv) host races, and (v) species (Table 1). Importantly, these categories produce predictable patterns of gene flow, selection, and genotypic differentiation within and between biotype populations (Table 1), allowing for straightforward hypothesis testing of biotype adaptation against the standard null hypotheses of no genetic differentiation and genetic isolation by geographic distance. Therefore, population genetic analysis should be among the initial steps in characterizing and describing biotype populations. As an example of the investigative power of the Diehl and Bush framework, we performed an empirical analysis of a recently discovered biotype in an invasive species, the soybean aphid.

**Table 1:** Hypotheses proposed through the Diehl and Bush (1984) framework with predicted population level patterns for each

		Predicted population level responses		
		
Diehl & Bush categories	Hypothesis	Gene flow	Structure	Genotypic diversity
Nongenetic	Biotypic differences are not genetic in origin, but are likely associated with phenotypic plasticity, environmental effects, and endosymbionts	Ubiquitous	No structure	Little to no deviation between biotypes. Shared *MLGs common
Ubiquitous genetic	Biotypes are the product of adaptive genetic variation, but gene flow is uninhibited. Biotypic traits subject to population level drift and selection	Ubiquitous	No structure	Limited deviation between biotypes if monogenic. None if polygenic
Geographic race	Biotypes are geographically separate during sexual stages, limiting gene flow. Biotypes evolved via geographic isolation	Restricted	Strong structure by biotype and geographic gradient	Deviation in richness between biotypes. Few Shared MLGs
Host race	Biotypes associate with different primary hosts causing near sexual isolation and divergent evolutionary trajectories	Restricted	Structure between biotypes and primary host	Deviation in richness between biotypes. Few Shared MLGs
Species	Biotypes are indicative of separate species that share no gene flow	None	Strong structure by biotype populations	Genotypes significantly divergent, few or no MLG shared between biotypes

* MLG, multilocus genotype.

### The soybean aphid

The soybean aphid (*Aphis glycines*) is a significant hemipteran pest of soybean (*Glycine max* L.) native to East Asia (Blackman and Eastop 1984). *A. glycines* has recently invaded North America and was first detected in Wisconsin soybean in 2000 (Wu et al. 2004; Ragsdale et al. 2011). Despite the presumed founder effect associated with invasion (Michel et al. 2009), the soybean aphid has proven to be well adapted to the industrial agro-ecosystem in North America and has rapidly expanded its range which includes 30 U.S. states and three Canadian provinces (Ragsdale et al. 2011). Within North America, the species is heteroecious and holocyclic, transitioning between asexual clonal reproduction on its secondary host, soybean, and a sexual phase on its primary and overwintering host (*Rhamnus* spp., with common buckthorn, *R. cathartica*, most frequently utilized) (Ragsdale et al. 2004). As a result of the ubiquitous presence of both primary and secondary hosts, and aphid movement among them, there are few barriers to gene flow among *A. glycines* populations, resulting in genetic homogenization across North America (Michel et al. 2009; Orantes et al. 2012).

Soybean aphid-resistant soybean cultivars have been developed to provide an alternative to comparably expensive and ecologically damaging chemical insecticides (Hodsgon et al. 2012). Currently, five *Rag* (Resistance to *Aphis glycines*) genes have been described, *Rag1 – Rag5* (Hill et al. 2006a,b; Mian et al. 2008; Zhang et al. 2008; Jun et al. 2012), with *Rag1* expressing varieties commercially released in 2010. Virulence to *Rag1* and *Rag2* has been found in natural soybean aphid populations; natural virulence to other *Rag* genes is unknown. Currently, four biotypes of the soybean aphid are recognized including: biotype 1 (not virulent, *i.e*., avirulent, to all HPR strains), biotype 2 (virulent only to *Rag1* soybean), and biotype 3 (virulent to *Rag2* while remaining mostly avirulent to *Rag1* soybean), and biotype 4 (virulent to Rag1 and Rag2 individually and in concert) (Kim et al. 2008; Hill et al. 2010; Alt and Ryan-Mahmutagic 2013).

Although much effort has been made to elucidate the mechanism and chromosomal location of aphid resistance within soybean (Li et al. 2008; Hill et al. 2012), relatively little effort has been focused on the ecological and evolutionary genetics of soybean aphid biotypes, including genetic comparisons between biotype populations. Considering the Diehl and Bush framework, virulent biotype evolution in *A. glycines* could be the result of any of the five described categories with patterns of genetic variability matching those listed in Table 1. However, the lack of population structure found in previous studies (Michel et al. 2009; Orantes et al. 2012) suggested that race formation and speciation are unlikely causes of biotypic virulence. Therefore, in this case, we predicted that biotypic virulence is a function of either nongenetic environmental influences or a genetic polymorphism that is ubiquitous throughout the population via sexual recombination.

To clarify the evolutionary genetic relationships among soybean aphid biotypes, we performed a molecular marker analysis of 14 populations of biotype 1 and 2, geographically distributed across seven collection sites in northern Ohio, USA. Genotypic diversity, genetic distance, and population assignment analyses were performed across geographies and biotypes. Our goals were to determine the pattern and level of genetic differentiation among biotype populations. If we find strong genetic structuring between biotype populations, then biotypes are the product of race formation or speciation through restricted gene flow. Alternatively, if structure is non-existent or unassociated with biotypes, then virulence is ubiquitous throughout the North American population and is genetically admixed or environmentally induced and nongenetic (Table 1). This is the first comparison of genetic variation between soybean aphid biotype populations, and the results of this study would lead to a better understanding the evolution of virulence and improve the use of resistant soybean cultivars.

## Materials and methods

### Collection and biotype differentiation

**Figure 1:**
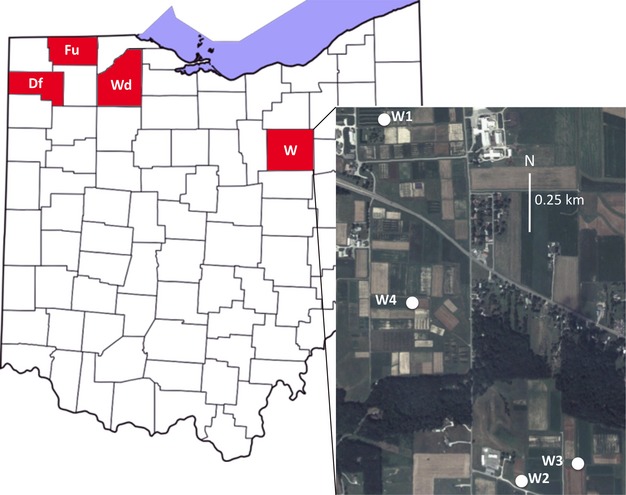
Geographic location of collections. Abbreviations and GPS coordinates available in Table 2.

Biotype 1 and 2 soybean aphids were sampled in pairwise, concurrent collections at seven sites across northern Ohio, with sites grouped into two geographic clusters (Fig. 1, Table 2). Distances between sites were designed to account for a null hypothesis of genetic isolation by geographic distance, with gradients representing three geographic scales termed microgeographic (<2 km), mesogeographic (>2 km, <120 km), and macrogeographic (>120 km). Site selection was based on affiliation with the Ohio State University extension system, available field space, and aphid infestation. The eastern cluster was composed of four sites at the Ohio Agricultural Research and Development Center (OARDC) in Wooster, Wayne County, Ohio; all Wayne county field sites were established within a 2 km radius to allow sampling on a microgeographic scale. The western cluster included single field sites in Defiance, Fulton, and Wood counties in northwest Ohio. The western cluster sites were located within 120 km of one another and 225 km from the eastern cluster, thereby establishing meso- and macrogeographic collection distances.

**Table 2:** Collection information for soybean aphid field sites

Field*	Collection date†	N-B1‡	N-B2‡	Latitude (N)	Longitude (W)
Defiance (Df)	September 1	45	48	41.324	84.551
Wood (Wd)	August 17	48	40	41.455	83.664
Fulton (Fu)	August 21	39	56	41.608	83.986
Wayne-1 (W1)	August 27–September 2	47	48	40.773	81.910
Wayne-2 (W2)	August 27–September 2	55	49	40.759	81.903
Wayne-3 (W3)	August 27–September 2	47	47	40.759	81.900
Wayne-4 (W4)	August 27–September 2	46	47	40.766	81.908

* Abbreviations in parentheses.

† All collections in year 2011.

‡ Sample size of biotype 1 (B1) and biotype 2 (B2).

Sampling of aphids differed between the western and eastern clusters due to growing space constraint within the eastern sites. Within these sites (W1-W4, Table 2), *Rag1* expressing LD-05 16060 (i.e., resistant soybean) and susceptible variety SD-01 76R (Tinsley et al. 2012) were grown in separate 38 × 53 cm growth flats. Soybeans were reared under standard greenhouse conditions until V3 growth stage and then transferred to the field sites. Single flats of both the *Rag1* and susceptible variety were positioned on the edge of a susceptible and insecticide-free soybean field. Plants were observed every 2 days for aphid colonization. Apterous (i.e., wingless) aphids collected in the field on *Rag1* expressing plants were assumed to be virulent and defined as biotype 2. Any aphids collected on susceptible soybean could be biotype 1 (avirulent to *Rag1*) or biotype 2 and therefore required further characterization using the detached leaf assay as outlined by Michel et al. (2010). Aphids capable of feeding and producing viable clones on the *Rag1* detached leaves were classified as biotype 2 and were excluded from our analysis. Aphids that failed to feed, actively avoided the leaf, produced unviable nymphs, or suffered mortality after feeding on the leaf were defined as biotype 1. All aphids were collected from their host plant using a fine tip brush and were stored at −20°C. For eastern cluster sites, no single daily collection produced enough aphids for population analysis; thus, collections were pooled (respective of geographic location and biotype) across an eight-day period of August 27 through September 2, 2012. These dates were selected as they coincide with peak aphid infestation in Ohio and overlap with northwestern cluster collection days. Furthermore, these dates were after known soybean aphid dispersal events that homogenize population structure, allowing us to avoid confounding geographic population structure associated with temporal factors (Orantes et al. 2012).

Within the western cluster sites (Df, Wd, Fu, Table 2), three rows of susceptible and *Rag1* expressing soybean were grown within insecticide-untreated susceptible soybean fields. Defiance (Df) was an exception, with three rows of soybean (per treatment) grown in a separate plot, not adjacent to conventionally grown soybean fields. At all locations, aphids were collected on a single day (Table 2) by collecting 50 infested leaves from both resistant and susceptible soybean. To avoid resampling genetically identical clones, a single leaf was collected from each sampled plant, with collected leaves stored in separate plastic bags. Leaves were then transported to the laboratory whereupon 1 aphid was removed from each leaf and stored at −20°C until later genetic analysis. Aphids from susceptible plants in the western cluster were subject to biotype determination via detached leaf assay as explained previously.

### DNA extraction and SNP genotyping

DNA was extracted from individual aphids using the QuickExtract Seed DNA Extraction Solution (Epicentre, Madison, WI, USA) per manufacturer's instructions. A total of 18 single nucleotide polymorphisms (Barker et al. 2011) were genotyped using the allele-specific primer extension (ASPE) technique, (Luminex, Austin, TX, USA), following the modified protocol outlined in Orantes et al. (2012). Initial isolation and amplification of genomic regions containing SNPs was performed with the Qiagen Multiplex PCR Kit (Qiagen, Valencia, CA, USA), with final product cleansing using ExoSAP-IT (Affymetrix Corporation, Santa Clara, CA, USA), following manufacturer's instructions. Samples were processed at the OARDC-Molecular and Cellular Imaging Center using the Luminex200 system. Allele calling was automated through the Masterplex QT and GT MiraBio program suites (San Francisco, CA, USA), with allele designations manually inspected and verified prior to statistical analyses.

### Neutrality and clonality

LOSITAN (Antao et al. 2008) was employed to assess selection neutrality at the individual loci. All loci found to be under directional or balancing selection were removed from the data set. Statistical analyses were performed on data sets both lacking and containing these loci to ascertain any biases.

While the sampling scheme was designed to avoid the sampling of clones, clonal identification was still required as to not bias the data set toward over-represented lineages (Arnaud-Haond et al. 2007). The data analysis programs GeneAlEx v. 6.41 (Peakall and Smouse 2006) and GenClone v. 2.0 (Arnaud-Haond and Belkhir 2007) were used to identify shared multilocus genotypes (MLGs, i.e., clones) within and between all populations. Within populations, all individuals with shared MLGs were removed so that only a single individual remained. Aphids with shared MLGs that were not within the same sampling location/biotype were retained. All statistical analyses were performed with data sets including and excluding shared MLGs, though the results presented exclude shared MLGs. GenClone was utilized to assess clonal lineage diversity and evenness within populations, a proxy measure of gene pool size and diversity. The Pareto distribution index (*C*) and the Simpson's diversity (*D*) and evenness score (*V*) were calculated to estimate the abundance and diversity of clones within and between the sampled populations. Additionally, all shared MLGs within a sampled population were assessed for origin of identity using GenClone's P_SEX_ statistic. P_SEX_ estimates the probability that individuals with a shared MLG were a product of sexual reproduction, as opposed to clonal propagation.

### Genetic diversity and structure

Hardy–Weinberg equilibrium, as measured through the inbreeding coefficient F_IS_, as well as expected and observed heterozygosity (*H*_E_ and *H*_O_, respectively), were calculated via GENEPOP (Raymond and Rousset 1995). *F*_IS_, *H*_O_, and *H*_E_ were compared across biotypes and eastern/western clusters using FSTAT V2.9.3 (Goudet 1995) with 10 000 random permutations. Linkage disequilibrium within populations was also assessed via FSTAT.

Population structure was analyzed through multiple analyses. MSA 4.05 (Dieringer and Schlötterer 2003) was used to calculate pairwise comparisons of the fixation index (*F*_ST_) between populations with Bonferroni-corrected *P*-values. Principal coordinate analysis (PCA) was performed using a Nei's pairwise distance matrix in GenAlEx v. 6.41. Population assignment was estimated using both the Paetkau assignment method (Paetkau et al. 2004) and STRUCTURE 2.3.3 (Falush et al. 2003) with 250 000 reps burnin and 750 000 reps analysis for each of five independent replications. Both assignment methods were assessed across biotypes and collection sites, both individually and grouped by biotype or geographic cluster.

## Results

### Loci neutrality

LOSITAN analysis revealed one locus under directional selection (42701) and two loci undergoing balancing selection (5109 and 2654) (Figure S1). The directional selection found in locus 42701 was geographically associated; the eastern cluster exhibited significantly higher heterozygosity (F_IS_: −0.726) than the populations within the western cluster (F_IS_: 0.015, FSTAT *P* < 0.001). Locus 42701 inflated *F*_ST_ values between the geographic clusters and was removed from the analysis. Loci 5109 and 2654 were not geographically or biotypically associated but were nonetheless removed from the data set to avoid confounding the patterns of divergence inherent within the remaining neutral markers. Among the remaining loci, limited linkage disequilibrium was detected between six loci, but was found to have no significant effect on statistical analyses or results.

### Clonal diversity

Analyzing the clonal diversity among biotypes can provide an indication of the prevalence of virulence in a population. If virulence is rare, then we would expect a small diversity of genotypes to be found on *Rag1* plants compared to susceptible plants due to the virulent biotype's restricted gene pool. However, clonal lineage assessment revealed multilocus genotype (MLG) diversity to be high both within and between biotype populations. Of the 662 sampled individuals, there were 575 distinct MLGs, of which 524 (91.1% total MLGs) were singletons (occurring in a single individual). Of the shared MLGs (51, 8.87% total MLGs), the majority were shared between individuals of the same biotype and collection site, while a minority were shared across biotype or site. P_SEX_ values for members of shared MLGs were significant (*P* < 0.05), indicative of clonal origin for shared genotypes. The most common shared MLG consisted of 18 biotype 2 individuals from the Df site. This population was also the least diverse genotypically, with 22 of its 48 sampled members (45.8%) belonging to a shared MLG. In contrast, biotype 2 from the Fu site had the greatest diversity of MLGs with only one aphid (1.8%) having a shared MLG.

Elevated genotypic diversity in many populations resulted in uninformative results for some MLG diversity statistics, as was seen in previous analyses of soybean aphid populations (Michel et al. 2009; Orantes et al. 2012). There was no decrease in MLG diversity within biotype 2 aphids when compared to biotype 1, suggesting that virulent aphid clones share a diverse sexual gene pool (Simpson's *D*: biotype 1 = 0.994, biotype 2 = 0.974, Wilcoxon: *P* > 0.2, *n* = 7, W = 12; Table 3). Simpson's evenness (*V*) was also not significantly different between biotypes (Wilcoxon, *P* > 0.2, *n* = 7, W = 4.96), averaging 0.566 for biotype 1, and 0.501 for biotype 2. These diversity measures remained nonsignificant when populations were compared across micro-, meso-, and macrogeographic distances (anova: Micro F(3,4) = 2.32, *P* > 0.2; Meso F(2,3) = 0.919, *P* > 0.2; Macro F(1,12) = 1.381 *P* > 0.2).

**Table 3:** Diversity statistics between soybean aphid biotypes

	Diversity statistics			Multilocus genotypes (MLGs)			
		
Site	Simpson *D*	Simpson V*	Pareto *C*†	Unique‡	Shared§	Clonal**	Total††
Biotype 1							
Df	0.997	0.512	<4.39	38	5	2	43
Wd	0.988	0.837	2.499	36	3	6	39
Fu	0.998	0.000	<5.25	38	0	1	38
W1	0.993	0.739	3.405	37	5	4	42
W2	0.986	0.795	2.446	41	3	6	44
W3	0.986	0.512	<4.43	42	3	2	45
W4	1.000	N/A	<1.00	45	1	0	46
Bio1 Avg	0.994	0.566	2.783	39.6	2.8	3	42.4
Biotype 2							
Df	0.835	0.281	1.26	28	0	4	28
Wd	0.991	0.801	3.304	33	1	5	34
Fu	1.000	N/A	<1.00	55	1	0	56
W1	0.998	0.511	<4.56	41	5	2	46
W2	0.995	0.639	3.531	41	4	3	45
W3	0.994	0.772	<3.44	40	3	3	43
W4	0.999	0.000	<5.426	41	5	1	46
Bio2 Avg	0.973	0.501	2.698	39.9	2.7	2.6	42.6

* N/A indicative of no shared MLG's within a population, complete diversity.

† ‘<‘ designate estimates of Pareto's C due to lack of MLG groups large enough for a complete estimate.

‡ Number of MLGs unique to the sampled population.

§ Number of MLGs within the sampled population that were shared with other populations.

** Number of shared MLGs within the sampled population that were due to clonal propagation.

†† Count of MLGs within the sample population (sum of Unique and Shared MLG totals).

### Genetic diversity and structure

Hardy–Weinberg disequilibrium was observed at several loci, with deviation occurring at 85 of a total possible 210 cases (40.5%). Total loci in disequilibrium per population averaged 6.07 (range 3–11). These results are greater than those observed in previous research (Orantes et al. 2012), though the number of deviations decreased when populations were grouped according to biotype or sampling cluster. This suggests that individual populations or yearly environmental conditions drive fluctuations in heterozygosity via clonal amplification rather than specific geographic or biotypic effects. Such dynamics are to be expected within an asexually reproducing and highly mobile organism. Overall, populations exhibited slightly more heterozygote excess (55%) than heterozygote deficiency (45%), with a mean *F*_IS_ = −0.018 among loci (Table S1). At the population level, *F*_IS_ deviations were typically slight and ranged from −0.114 in Df biotype 1 to 0.062 in Wd biotype 2. Deviation between biotypes was nonsignificant in F_IS_ and heterozygosity (expected or observed). Similarly, no differences in *H*_*O*_ or *F*_IS_ were observed when grouped by eastern and western clusters; however, there was significant difference in expected heterozygosity (FSTAT, 10 000 permutations, *P* < 0.05) between the clusters due to a relatively high proportion of homozygotes in both biotype populations from the Wd site.

Population pairwise *F*_ST_ was generally low and ranged between −0.008 and 0.064 with a mean value of 0.013 (Table S2). No populations were found to have a significantly different pairwise F_ST_ values other than Df, where differences were not associated with geography or biotype. With the exception of the Df field, aphids of different biotype collected at the same site did not show significant genetic differentiation. Similar results were seen after pooling within biotypes (F_ST_ = −0.005, *P* > 0.68). However, significant differences were found when populations were compared among and between meso- and macrogeographic distance, with Df showing significant difference from Fu (F_ST_ = 0.011, *P* < 0.05) and W1-4 sites (F_ST_ = 0.009, *P* < 0.01).

**Figure 2:**
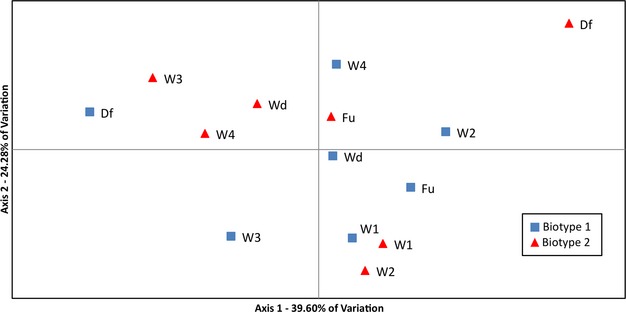
Principle Components Analysis (PCA) of Nei's Genetic Distance between biotypes 1 and 2 of sampled populations. No discernible patterns are found between biotypes or geographic distance, but rather patterns indicative of panmixia.

If genetic variation in soybean aphid populations was associated with either biotypes or geography, then the PCA would have reflected nonrandom clustering respective of either of these two factors (Figure S2A,B). Instead, PCA using Nei's genetic distance did not reveal any obvious pattern associated with geographic location or biotype (Fig. 2). The PCA further suggests that while the W1-4, Fu, and Wd populations of both biotypes are genetically similar, both Df biotype populations are strongly divergent from the other sites and each other. Population assignment tests lend further support to this unstructured pattern. Paetkau self-assignment consistently assigned the majority of individuals within each population as migrants (mean 88%; range 55%–100%, Table 4), suggesting high mobility of individuals. Likely due to this immigration, STRUCTURE analysis failed to detect any significant population structure aside from *k* = 1. Both the STRUCTURE and Peaktau analyses suggest little genetic differentiation between populations according to biotype or geographic location.

**Table 4:** Population assignment of individual MLGs within their sampled populations via Paetkau assignment test

	Paetkau assignment		
	
	Self	Out	% Self*
Biotype 1			
Df	15	28	34.9
Wd	0	42	0
Fu	1	37	2.6
W1	2	41	4.6
W2	3	41	6.8
W3	4	41	8.9
W4	7	39	15.2
B1 Avg	4.5	38.4	10.4
Biotype 2			
Df	13	16	44.8
Wd	0	34	0
Fu	8	48	14.3
W1	2	44	4.3
W2	5	42	10.6
W3	8	35	18.6
W4	2	44	4.4
B2 Avg	5.4	37.5	13.9

* Percent of population assigned to the original collection.

## Discussion

In this study, we applied a population genetic framework to investigate the genetic relationship among virulent and avirulent insect biotypes. Utilizing the Diehl and Bush categories of biotype evolution, we have constructed a five part hypothetical framework along with predictive responses for each category (Table 1). The lack of significant patterns of genetic differentiation between biotypes or across sites suggests that the virulent phenotype is both widely distributed within the North American population and readily admixed during sexual reproduction. These patterns are consistent with the predicted population responses of nongenetic or ubiquitously dispersed genetic mechanisms of biotypic virulence.

### Extensive genetic homogeneity between biotypes and geography

Population genetic analysis of soybean aphid biotypes 1 and 2 showed that there is no significant differentiation between populations segregated by biotype or geographic location, but rather was consistent with genetic homogeneity across the landscape (Tables 3 and 4, Fig. 2). The results are in agreement with previous population genetic analyses of the soybean aphid (Michel et al. 2009; Orantes et al. 2012), which suggested population structure within North America is limited by late season dispersal of unique MLGs across the Midwestern US—a landscape dominated by agroecosystems and large availability of suitable habitat. We did observe slightly higher levels of HWE deviation (as compared to Orantes et al. 2012), from both heterozygote excess and deficiency. However, the number of loci that deviated from HWE decreased when populations were grouped according to biotype or sampling cluster, suggesting that individual populations or yearly environmental events drive fluctuations in heterozygosity via clonal amplification rather than any specific effects of geography or biotype.

Contrary to these general results, both biotype populations from the Df site were genetically distinct from one another and other populations (Table S2). Indeed, the highest F_ST_ value in the study was not among the most geographically distant sites, but between the biotype populations in Df (F_ST_ = 0.064, *P* < 0.05). The unusually large genetic differentiation between these populations was not due to genotyping error, systematic artifacts, or sampling bias. Rather, the source of the deviation was likely environmental, due to the unique growing conditions at this collection site. Unlike the other populations where the sampled plants were embedded in larger production fields, the sampled plants in Df were instead grown in separate rows, relatively isolated from nearby suitable *A. glycines* habitat. Additionally, Defiance County experienced an unusually dry and warm July, with mean high temperatures consistently >32°C. At this temperature, soybean aphids are known to suffer elevated mortality at the first instar (58% survival) (Hirano et al. 1996). We infer that a relatively small colonization of soybean occurred due to the small area of the plants and isolation from surrounding fields. After colonization, the Df sites suffered significant mortality, and any remaining clones were restricted in their reproductive capacity due to environmental conditions. When conditions were more conducive for aphid growth, the few remaining clones were able to rapidly reproduce and spread within the plot, becoming dominant MLGs within population. For example, 3 MLGs accounted for 46% of the individuals collected in the biotype 2 from Df. In our larger sites more consistent with typical agronomic conditions, we did not observe a decrease in clonal diversity or significant genetic differentiation among biotypes. Therefore, isolated fields similar to the Df site could lead to the development of biotype-specific MLGs, but these aphids will be unlikely to find suitable overwintering hosts or be vastly outnumbered by migrants from larger and more conventional surrounding fields (Orantes et al. 2012).

### Application of the Diehl Bush framework

The Diehl and Bush framework divides biotypes into five possible evolutionary categories: (i) nongenetic polymorphism, (ii) polymorphic or polygenic variation within populations, (iii), geographic races, (iv) host races, and (v) species (Table 1). These categories are differentiable through the population structure, gene flow, and genotypic diversity patterns they project onto the genetic variation of sampled populations. Our data did not show evidence of genetic structuring between biotypes (Figure S2A) or geographic location of sampling sites (Figure S2B), which would rule out (iii) geographic races, (iv) host races, and (v) species. This lack of structure appears to be associated with widespread gene flow between populations during the sexual stage due to elevated interpopulation migration (Table 4). When compared to the predictive Diehl and Bush framework (Table 5), our results suggest two hypothetical virulence mechanisms: (i) a nongenetic source and (ii) a ubiquitously dispersed and sexually admixed genetic source (Table 5).

**Table 5:** Predicted patterns of gene flow, population structure, and genotypic diversity per Diehl & Bush (1984) category and sub-category. Categories in bold are consistent with data in the present study

Diehl & Bush category	Sub-category	Genetic differentiation	Population structure	Genotypic diversity
Nongenetic	Endosymbiont	Between biotype	Minor structure biotype or geography	Biotype 2 less diverse
**Nongenetic**	**Phenotypic Plasticity**	**None**	**No clustering or by geography**	**No significant difference between biotypes**
**Ubiquitous genetic**	**Gene for Gene**	**None**	Minor structure biotype or geography	Biotype 2 less diverse
**Ubiquitous genetic**	**Epistasis**	**None**	**No clustering or by geography**	**No significant difference between biotypes**
Geographic race	N/A	Strong between biotype	Cluster by biotype	Sig Dif in diversity
Host race	N/A	Strong between biotype	Cluster by biotype	Significant difference in diversity
Species	N/A	Strong between biotype	Cluster by biotype	Significant difference in diversity

### Possible nongenetic sources of virulence

Nongenetic sources include the presence of phenotypic plasticity; phenotypes are environmentally dependent provided enough genetic variation is present in the population to produce a large phenotypic range (i.e., the norm of reaction). In this case, genetic variation between biotypes is expected to be minimal (Tables 1 and 5), the primary pattern found within our data. Additionally, some MLGs were shared between biotypes at a sampling site, suggesting that the divergent virulent phenotype is being expressed differentially across individuals of the same clonal lineage. Many aspects of the soybean aphid's life history make the species particularly capable of nongenetic adaptation including endosymbiont associations, telescoping generations, and cyclical asexuality (Dixon 1985; Moran 1992).

Many insect species, particularly aphids, are known to harbor bacterial endosymbionts. These associations can be either obligatory or facultative and provide insects with selective advantages such as nutritional supplements, host specificity, and defense against environmental stressors (Moran et al. 2008; Douglas 2009; Oliver et al. 2010). The soybean aphid is known to harbor three endosymbionts: the obligate *Buchnera aphidicola* and two facultative species: *Wolbachia* and *Arsenophonus* (Liu et al. 2012). *B. aphidicola* is required for the isolation and synthesis of essential amino acids from the aphid's nitrogen poor diet of phloem sap (Baumann et al. 1995; Douglas 1998, 2009). *Rag1* plants have altered ratios of free amino acids in soybean leaves compared to non-*Rag1* plants (Chiozza et al. 2010) and may select for a *B. aphidicola* strain(s) able to overcome the deficient amino acid content. *B. aphidicola* upregulated different proteins when their potato aphid (*Macrosiphum euphorbiae)* host was reared on diverse resistant cultivars (Francis et al. 2010), suggesting an adaptive role. However, if *B. aphidicola* strains were responsible for virulence in biotype 2, then genetic differentiation should have been apparent, as the symbiont is passed maternally and the resulting virulent offspring would be closely related.

Facultative endosymbionts are associated with a number of traits in aphids including nutrition, heat resistance, parasitoid immunity, and host-plant transitions (Oliver et al. 2010), but the role of *Arsenophonus* and *Wolbachia* in the soybean aphid is unclear. Wulff et al. (2013) found *Arsenophonus* to be harbored in 80 of 83 sampled North American *A. glycines*, though it did not provide protection against parasitoids or the fungus *Pandora neoaphidis*. Additionally, there is currently little or no evidence of facultative symbionts being involved in biotypic virulence. A preliminary PCR screening of *Arsenophonus* across 128 randomly selected aphids from our data set revealed the symbiont to be almost universally present across site and biotype (data not shown). Future studies are needed to understand the interaction between the soybean aphid and its various endosymbionts.

In addition to potential endosymbiont effects, aphids are known for their ability to express widely divergent phenotypes within a genetically identical clonal lineage, such as the ability to transition between asexual and sexual forms as well as winged and nonwinged forms within a single asexual generation (Moran 1992; Via 1993; Weisser and Stadler 1994). If plasticity is involved, then virulence may not be static or restricted to certain genotypes; instead, formerly avirulent aphids may express virulence to resistant cultivars in response to appropriate environmental stimuli. Soybean aphids exhibit variation in morphological and reproductive traits in response to elevated temperature and can increase clonal fecundity as asexual generations progress under detrimental conditions (Richardson et al. 2011). This suggests adaptive phenotypic plasticity is possible in response to environmental change, a mechanism that could play a role in biotypic virulence through differential gene regulation. Additionally, multitrophic interactions between the aphid, host plant, and environmental variables are likely to facilitate virulence, as the level of resistance in certain soybean cultivars can be affected by temperature (Richardson 2011). In this case, virulence is not necessarily wholly dependent on the aphid's genotype, but the environmental context in which resistance is manifested.

Performance on resistant soybean cultivars may also be explained by prior host exposure (Schotzko and Smith 1991; Robinson 1993). When exposed to resistant sorghum for 24 h, Greenbugs (*Schizaphis graminum*) were found to locate phloem faster and feed longer than individuals previously unexposed to the resistant plant, implying adaptive plasticity (Montllor et al. 1983). Similarly, soybean aphids not initially repulsed by resistant soybean may alter their behavior on the plant, allowing for greater fitness over time. Conditioning may also occur on different species of host plants. For example, *A. fabae* clones exhibit differences in host-plant preference due to initial rearing plant (Gorur et al. 2007), and *A. gossypii* fed on different host plants develop differential morphologies (Wool and Hales 1997). Although the soybean aphid has not been found to form colonies on alternative primary hosts, it can temporarily feed on other plants during migration events (Alleman et al. 2002). An intriguing hypothesis, then, is that feeding on these alternative hosts en route to soybean may prime the aphid for survival on resistant soybean and possibly result in biotypic virulence.

### Ubiquitous genetic sources of virulence

The ubiquitous genetic source differentiates itself from race formation and speciation in that it is not the product of divergent evolution through isolation, but rather is indicative of adaptive genetic variation that is dispersed throughout a population. In such a case, little or no genetic differentiation would then be observed between the diverging phenotypes (Table 1), a prediction supported by our data (Table 5). Genetic virulence is not unexpected, as the *Rag1* cultivar was obtained through interlineage crossbreeding (Hill et al. 2006a), and soybean aphids are likely to have experienced coevolution with variants of the *Rag1* gene. Thus, virulence traits could have pre-existed within the invasive population and then became widely dispersed.

Biotypic virulence is commonly explained via the gene-for-gene model, in which plant resistance and biotypic dynamics are characterized by interactions between gene pairings within the interacting species (Smith and Boyko 2007; Harris et al. 2012; Smith and Clement 2012). Aphids are known to produce effectors, small proteins that modulate plant host cell and defense processes (Hogenhout et al. 2009; Bos et al. 2010), which may provide genetic machinery for such a gene-for-gene response. Without selection pressure from widely planted resistant crops, virulent biotypes may exist at a low frequency. In this situation, the gene-for-gene model is likely to display some level of selective signature within the population, as virulent genotypes would be drawn from a limited gene pool. This pattern was not found within our data, where MLG diversity was high and statistically identical between biotypes (Table 3). Additionally, several MLGs were shared across biotypes at a geographic location, suggesting individuals of the same clonal lineage can express both the virulent and avirulent phenotypes. These data are suggestive of both phenotypes drawing from similarly sized gene pool with no virulence specific genotype.

An alternative to the gene-for-gene model is that biotypic virulence is an epistatic trait, referred to as ‘complex polygenic mechanism(s)’ by Diehl and Bush (1984). The epistatic model presumes multiple loci bear alleles providing partial virulence that act synergistically to amplify an individual gene's adaptive potential when held in combination. Through recombination, these adaptive alleles are mixed and may occur in multiple, potentially favorable, combinations. In such a model, the inheritance of partial complexes could lead to marginal virulence, and a gradient of fitness on *Rag1* soybean across aphid genotypes. Such a gradient has been observed in field collected aphids reared on detached *Rag1* soybean leaves (Michel et al. 2010, 2011). Furthermore, minor QTLs associated with resistance are not uncommon in soybean cultivars (Zhang et al. 2008; Jun et al. 2012), which could provide an avenue for an epistatic response.

## Conclusions

Population genetic analysis of soybean aphid biotypes has uncovered no significant genetic differentiation across either geographic space or biotypic designation. Biotypic virulence appears to be associated with a nongenetic source or genetic mechanism combined with uninhibited gene flow, dispersal, and sexual recombination. Environmental variables are known to affect both soybean resistance and aphid physiology, suggesting that biotypic virulence may be the result of phenotypic plasticity through multitrophic interactions. However, the current lack of information regarding North American *A. glycines* ecology and functional genetics provides challenges to understanding biotype evolution. Furthermore, as the use of resistant soybean increases, we may see more dramatic shifts in virulence frequency not revealed through this study using a single collection year. Future research investigating the role of endosymbionts, phenotypic plasticity, gene complexes, and their synergistic interaction with host plants and environmental variables is required to isolate the specific mechanism of virulence. Understanding the evolutionary and ecological mechanisms of insect adaptation to resistant hosts will be instrumental in the development of resilient insect resistance management programs.

## Data archiving statement

Data for this study are available in Dryad: doi:10.5061/dryad.092fc.

## Supporting Information

**Table S1.** Polymorphism statistics of loci per population, and HWE significance.

**Figure S1.** LOSITAN output from the complete loci dataset with clones removed.

**Figure S2.** Hypothetical PCA indicative of: (A) Predicted pattern of biotypic clustering associated with restricted gene flow of race formation and speciation. (B) Predicted null pattern of isolation by distance predicted in systems with limited gene flow, but limited selection.
