# Supplementary material for: Implementing an evolutionary framework for understanding genetic relationships of phenotypically defined insect biotypes in the invasive soybean aphid (Aphis glycines)
Source: Evol Appl. 2013 Jul 24;6(7):1041–53. doi: 10.1111/eva.12084 (PMC3804237; doi:10.1111/eva.12084)
Supplement: Supplementary file 1 [file eva0006-1041-SD1.pdf]

Supplemental Information

Table S1: Polymorphism statistics of loci per population, and HWE significance.

Figure S1: LOSITAN output from the complete loci dataset with clones removed. Locus 42701 exhibits significant directional selection. 5109 and 2654 show significant balancing selection.

Figure S2: Hypothetical PCA indicative of: A) Predicted pattern of biotypic clustering associated with restricted gene flow of race formation and speciation. B) Predicted null pattern of isolation by distance predicted in systems with limited gene flow, but limited selection.

Supplemental Materials

Table S1: Polymorphism Statistics of loci per population, and HWE significance.

	Df-B1				Df-B2			
**Locus**	H_O	H_E	F_{IS}	**HWE**	H_O	H_E	F_{IS}	**HWE**
**20935**	0.302	0.447	0.324	*	0.103	0.490	0.789	***
**13475**	0.395	0.369	-0.071	ns	0.621	0.499	-0.243	ns
**3383**	0.651	0.439	-0.483	**	0.448	0.383	-0.169	ns
**7006**	0.535	0.392	-0.365	*	0.483	0.366	-0.318	ns
**4773**	0.488	0.496	0.015	ns	0.214	0.375	0.429	*
**2836**	0.143	0.245	0.417	**	0.138	0.428	0.678	***
**47077**	0.907	0.496	-0.830	***	0.690	0.452	-0.526	**
**11160**	0.500	0.466	-0.074	ns	0.607	0.499	-0.216	ns
**934**	0.721	0.496	-0.454	**	0.345	0.366	0.058	ns
**25779**	0.000	0.499	1.000	***	0.000	0.245	1.000	***
**26668**	0.535	0.412	-0.297	ns	0.690	0.471	-0.465	*
**1708**	0.349	0.412	0.154	ns	0.655	0.498	-0.317	ns
**37780**	0.581	0.473	-0.229	ns	0.414	0.452	0.084	ns
**8466**	0.907	0.500	-0.814	***	0.897	0.499	-0.795	***
**2816**	0.302	0.317	0.047	ns	0.250	0.219	-0.143	ns
	**Wd-B1**				**Wd-B2**			
**20935**	0.262	0.466	0.438	**	0.176	0.472	0.626	***
**13475**	0.595	0.436	-0.365	*	0.412	0.472	0.128	ns
**3383**	0.595	0.436	-0.365	*	0.529	0.457	-0.159	ns
**7006**	0.524	0.387	-0.355	*	0.529	0.389	-0.360	*
**4773**	0.195	0.476	0.590	***	0.294	0.498	0.410	*
**2836**	0.220	0.457	0.520	***	0.118	0.498	0.764	***
**47077**	0.929	0.497	-0.867	***	0.824	0.484	-0.700	***
**11160**	0.690	0.500	-0.382	*	0.618	0.500	-0.236	ns
**934**	0.452	0.452	-0.001	ns	0.529	0.500	-0.059	ns
**25779**	0.024	0.486	0.951	***	0.029	0.465	0.937	***
**26668**	0.452	0.477	0.052	ns	0.353	0.360	0.019	ns
**1708**	0.571	0.427	-0.337	*	0.441	0.489	0.098	ns
**37780**	0.524	0.427	-0.225	ns	0.647	0.457	-0.417	*
**8466**	0.643	0.452	-0.422	**	0.853	0.496	-0.719	***
**2816**	0.286	0.337	0.152	ns	0.235	0.291	0.190	ns
	**Fu-B1**				**Fu-B2**			
**20935**	0.474	0.488	0.028	ns	0.607	0.448	-0.354	**
**13475**	0.553	0.472	-0.171	ns	0.179	0.392	0.545	***
**3383**	0.632	0.478	-0.322	*	0.446	0.500	0.107	ns
**7006**	0.342	0.284	-0.206	ns	0.375	0.305	-0.231	ns
**4773**	0.395	0.441	0.106	ns	0.536	0.490	-0.094	ns
**2836**	0.526	0.478	-0.101	ns	0.661	0.492	-0.342	*
**47077**	0.703	0.456	-0.542	***	0.839	0.487	-0.723	***
**11160**	0.289	0.375	0.228	ns	0.375	0.416	0.098	ns

**934**	0.500	0.441	-0.133	ns	0.464	0.494	0.061	ns
**25779**	0.026	0.458	0.943	***	0.036	0.436	0.918	***
**26668**	0.447	0.458	0.023	ns	0.429	0.448	0.044	ns
**1708**	0.632	0.450	-0.403	*	0.536	0.490	-0.094	ns
**37780**	0.526	0.411	-0.279	ns	0.607	0.459	-0.322	*
**8466**	0.605	0.441	-0.371	*	0.893	0.494	-0.806	***
**2816**	0.289	0.317	0.086	ns	0.339	0.347	0.022	ns
	**W1-B1**				**W1-B2**			
**20935**	0.452	0.452	-0.001	ns	0.457	0.415	-0.101	ns
**13475**	0.535	0.431	-0.242	ns	0.413	0.447	0.076	ns
**3383**	0.442	0.431	-0.026	ns	0.478	0.423	-0.129	ns
**7006**	0.395	0.344	-0.148	ns	0.435	0.340	-0.278	ns
**4773**	0.442	0.447	0.011	ns	0.370	0.415	0.109	ns
**2836**	0.571	0.500	-0.143	ns	0.630	0.494	-0.276	ns
**47077**	0.767	0.473	-0.623	***	0.652	0.440	-0.484	**
**11160**	0.349	0.473	0.262	ns	0.391	0.440	0.110	ns
**934**	0.548	0.497	-0.101	ns	0.565	0.485	-0.166	ns
**25779**	0.073	0.489	0.850	***	0.065	0.494	0.868	***
**26668**	0.442	0.483	0.085	ns	0.587	0.471	-0.245	ns
**1708**	0.419	0.422	0.008	ns	0.413	0.447	0.076	ns
**37780**	0.395	0.392	-0.009	ns	0.500	0.415	-0.206	ns
**8466**	0.837	0.487	-0.720	***	0.609	0.466	-0.306	*
**2816**	0.302	0.344	0.122	ns	0.239	0.375	0.362	*
	**W2-B1**				**W2-B2**			
**20935**	0.477	0.442	-0.080	ns	0.404	0.477	0.153	ns
**13475**	0.500	0.499	-0.002	ns	0.447	0.467	0.044	ns
**3383**	0.409	0.416	0.017	ns	0.489	0.409	-0.195	ns
**7006**	0.432	0.339	-0.275	ns	0.426	0.335	-0.270	ns
**4773**	0.500	0.474	-0.054	ns	0.383	0.435	0.119	ns
**2836**	0.500	0.500	-0.001	ns	0.326	0.481	0.322	*
**47077**	0.619	0.427	-0.448	**	0.702	0.456	-0.541	***
**11160**	0.455	0.474	0.041	ns	0.565	0.454	-0.246	ns
**934**	0.409	0.474	0.137	ns	0.468	0.500	0.063	ns
**25779**	0.045	0.416	0.891	***	0.064	0.496	0.871	***
**26668**	0.545	0.483	-0.128	ns	0.404	0.427	0.053	ns
**1708**	0.477	0.500	0.045	ns	0.447	0.456	0.019	ns
**37780**	0.455	0.416	-0.092	ns	0.511	0.400	-0.276	ns
**8466**	0.864	0.491	-0.760	***	0.809	0.489	-0.654	***
**2816**	0.386	0.339	-0.141	ns	0.255	0.282	0.096	ns
	**W3-B1**				**W3-B2**			
**20935**	0.511	0.452	-0.132	ns	0.465	0.467	0.005	ns
**13475**	0.356	0.429	0.171	ns	0.419	0.467	0.104	ns
**3383**	0.578	0.444	-0.300	*	0.395	0.490	0.194	ns
**7006**	0.400	0.320	-0.250	ns	0.395	0.317	-0.246	ns
**4773**	0.167	0.486	0.657	***	0.162	0.470	0.655	***

**2836**	0.182	0.491	0.629	***	0.175	0.475	0.631	***
**47077**	0.689	0.452	-0.525	***	0.698	0.454	-0.536	***
**11160**	0.378	0.464	0.187	ns	0.548	0.497	-0.101	ns
**934**	0.667	0.498	-0.339	*	0.605	0.498	-0.215	ns
**25779**	0.089	0.494	0.820	***	0.047	0.478	0.903	***
**26668**	0.556	0.437	-0.272	ns	0.535	0.412	-0.297	ns
**1708**	0.422	0.401	-0.052	ns	0.535	0.461	-0.160	ns
**37780**	0.622	0.458	-0.358	*	0.535	0.490	-0.091	ns
**8466**	0.844	0.488	-0.731	***	0.930	0.498	-0.870	***
**2816**	0.289	0.333	0.133	ns	0.512	0.381	-0.344	*
	**W4-B1**				**W4-B2**			
**20935**	0.370	0.352	-0.049	ns	0.304	0.315	0.033	ns
**13475**	0.435	0.440	0.011	ns	0.217	0.405	0.464	**
**3383**	0.378	0.491	0.231	ns	0.511	0.437	-0.170	ns
**7006**	0.364	0.298	-0.222	ns	0.391	0.315	-0.243	ns
**4773**	0.130	0.496	0.737	***	0.200	0.484	0.587	***
**2836**	0.419	0.487	0.140	ns	0.364	0.463	0.214	ns
**47077**	0.667	0.444	-0.500	***	0.689	0.452	-0.525	***
**11160**	0.489	0.444	-0.100	ns	0.432	0.479	0.099	ns
**934**	0.326	0.460	0.291	*	0.370	0.481	0.231	ns
**25779**	0.022	0.396	0.945	***	0.087	0.499	0.826	***
**26668**	0.587	0.471	-0.245	ns	0.674	0.460	-0.465	**
**1708**	0.609	0.485	-0.255	ns	0.370	0.447	0.173	ns
**37780**	0.565	0.476	-0.187	ns	0.457	0.447	-0.022	ns
**8466**	0.848	0.488	-0.736	***	0.935	0.498	-0.878	***
**2816**	0.391	0.315	-0.243	ns	0.543	0.396	-0.373	*

H_O: Observed Heterozygosity, H_E: Expected Heterozygosity, F_{IS}: Inbreeding Coefficient = $(H_E-H_O)/H_E$, HWE: Significance test for deviation from Hardy-Weinberg Equilibrium ns=Non-Significant Deviation, asterisks denote significant deviation with * ($p<0.05$), ** ($p<0.01$), *** ($p<0.001$). Significance determined via GenAlEx 2.0.

Table S2: Pairwise population comparison F_{st} values. All significant F_{st} scores in bold ($p<0.05$).

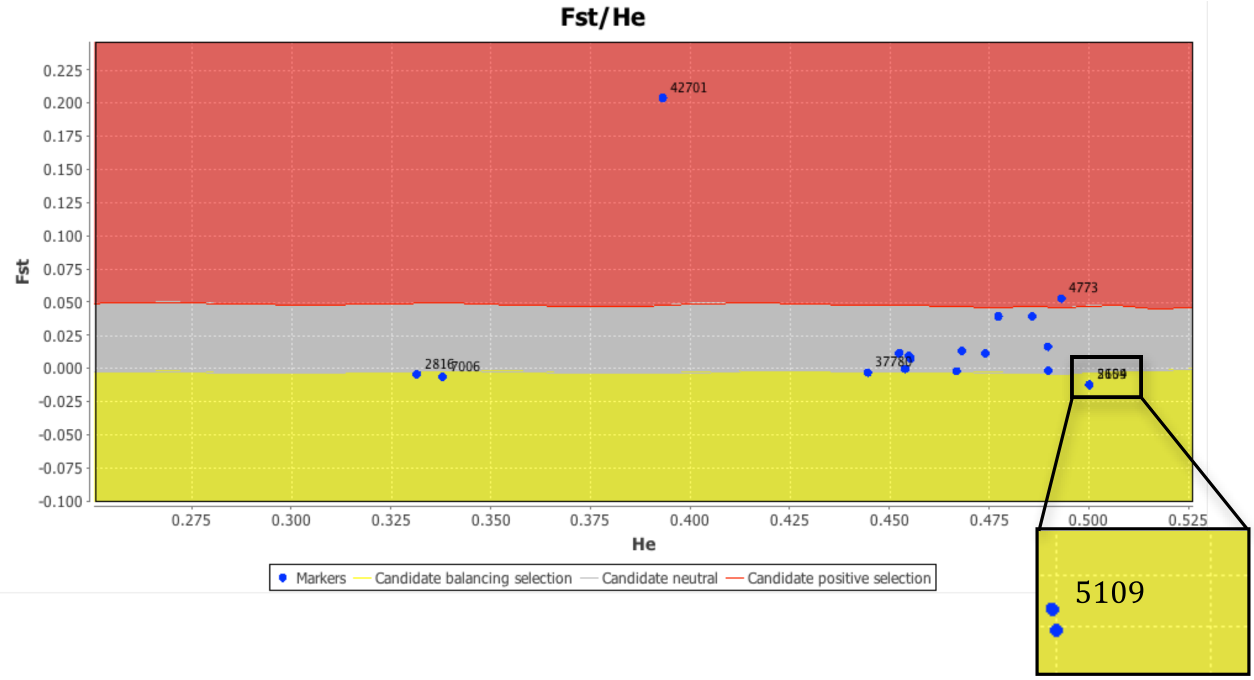

Figure S1: LOSITAN output from the complete loci dataset with clones removed. Locus 42701 exhibits significant directional selection. 5109 and 2654 show significant balancing selection.

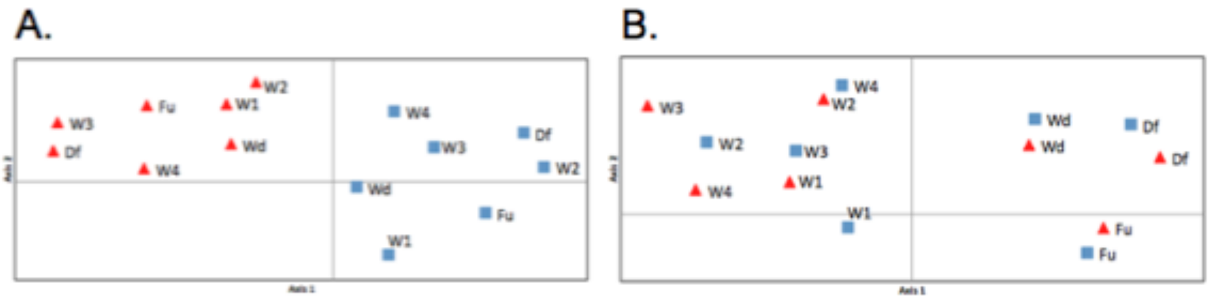

Figure S2: Hypothetical Principal Component Analyses indicative of: A) Predicted pattern of biotypic clustering associated with restricted gene flow of race formation and speciation. B) Predicted null pattern of isolation by distance predicted in systems with limited gene flow, but limited selection.
